# *Lactiplantibacillus plantarum* monolayer enhanced bactericidal action of carvacrol: biofilm inhibition of viable foodborne pathogens and spoilage microorganisms

**DOI:** 10.3389/fmicb.2023.1296608

**Published:** 2023-11-22

**Authors:** Valeria Poscente, Luciana Di Gregorio, Manuela Costanzo, Chiara Nobili, Roberta Bernini, Luigi Garavaglia, Annamaria Bevivino

**Affiliations:** ^1^Department for Sustainability, Biotechnologies and Agroindustry Division, Italian National Agency for New Technologies, Energy and Sustainable Economic Development (ENEA), Rome, Italy; ^2^Department of Agriculture and Forest Sciences, University of Tuscia, Viterbo, Italy; ^3^I.L.P.A. Group, Valsamoggia, Italy

**Keywords:** biofilm control, carvacrol, pre-formed biofilm, probiotics, flow cytometry, food spoilage, foodborne pathogens, food packaging

## Abstract

The prevalence of biofilm-associated microorganisms and the increasing use of ready-to-eat fresh products represent the current duality the food industry must address. Innovative and eco-friendly antibiofilm solutions and appropriate microbiological food control systems are urgently needed to improve food quality and safety. This study aimed to investigate the *in vitro* combined efficacy of carvacrol with a pre-formed biofilm monolayer of the probiotic *Lactiplantibacillus plantarum* DSM 20174. The antimicrobial activity of carvacrol against both planktonic and sessile cells of foodborne pathogens and spoilage microorganisms, alone or in the presence of the pre-formed biofilm of *L. plantarum*, was investigated by culture-based methods along with flow cytometry (FCM) to monitor cells' cultivability and viability. The synergistic action of carvacrol and the pre-formed biofilm of *L. plantarum* was evaluated in the 96-well plates. The results showed that *L. plantarum* pre-formed biofilm monolayer enhanced the antimicrobial effect of carvacrol determining a bactericidal action while the treatment alone induced the viable but not culturable (VBNC) cell state only. Furthermore, the great efficacy of the combined treatment allowed the application of a lower concentration of carvacrol (100 ppm) to achieve significant damage in cell viability. In conclusion, the incorporation of carvacrol into the *L. plantarum* pre-formed biofilm represents a promising alternative for an antimicrobial functionalized ready-to-eat packaging.

## 1 Introduction

The increasing focus on packaged and ready-to-eat fresh products has heightened the risks associated with the prevalence of biofilm-forming microorganisms and the limited effectiveness of current antimicrobial compounds, resulting in the urgent need to develop innovative and eco-friendly antibiofilm agents and appropriate food microbiological monitoring and control systems, combining concepts of quality, safety, sustainability, and consumer acceptability (Bondi et al., 2014; Di Carli et al., 2015; Tseng et al., 2022; Khodaei et al., 2023). Biofilms are formed by micro-structured communities of microorganisms embedded in a self-produced adhesive and protective extracellular polymeric substance (EPS), showing variations in growth rate and gene expression compared with their planktonic form (Asare et al., 2022). There is ample evidence from the scientific literature that bacteria in the biofilm phenotype are more resistant to antimicrobial agents than their planktonic counterparts due to the EPS matrix cell protection (Natan and Banin, 2017; Fulaz et al., 2019; Asare et al., 2022). In the food industry, microorganisms can adhere to packaging surfaces, form a biofilm, and compromise the shelf-life of food products (Galié et al., 2018; Carrascosa et al., 2021). In addition, studies show that bacteria in a biofilm state are more likely disposed to enter in the “viable but non-culturable” (VBNC) condition, where the cells are unable to grow and replicate on standard solid culture media, eluding detection by using conventional microbial culture-based techniques (Li and Zhao, 2020; Qi et al., 2022; Zhang et al., 2023). In this scenario, flow cytometry (FCM) emerges as a real-time technique for identifying the culture-uninvestigable cellular physiological state. The use of selected probes able to monitor several cellular parameters (i.e., membrane permeability, metabolic activity, and DNA/RNA content) enables obtaining a deeper knowledge of the mechanisms behind microbial inactivation and related subpopulation viability state response on both planktonic and sessile cells (March-Rosselló, 2017; Arioli et al., 2019; Truchado et al., 2020; Zand et al., 2021). Among new technological approaches in food packaging, the application of natural compounds as essential oils (EOs) and their components have been increasing in the last few years, to guarantee safety and preserve nutritional and organoleptic characteristics of food, in line with the “green and aware consumer” demand (Ojogbo et al., 2020; Abers et al., 2021; Cano et al., 2021; Angane et al., 2022). Particularly, essential oils of different *Origanum* species have exhibited antimicrobial effects against several bacteria, such as *Escherichia coli, Pseudomonas* spp., *Staphylococcus aureus, and Salmonella* spp. and fungi, including *Candida albicans*, and *Aspergillus* spp. Their efficacy can be attributed to the synergy of compounds such as carvacrol, thymol, and γ-terpinene, as well as *cis*- and *trans*-sabinene hydrate, with effective antimicrobial activity (Chouhan et al., 2017; Fikry et al., 2019; Angane et al., 2022). Biofilm control strategies are increasingly focused on the utilization of natural compounds and, particularly, carvacrol, for their capacity to selectively target the biofilm signaling pathways that regulate quorum sensing (QS), extracellular polymeric substance (EPS) synthesis, biofilm-related gene expression, microbial motility, adhesion, and dispersion. These findings are encouraging as they present a cost-effective, naturally derived anti-biofilm agent without inducing harmful side effects (Burt et al., 2014; Asma et al., 2022; Lu et al., 2022). Carvacrol, cymophenol (2-methyl-5-propan-2-yl-phenol), is a monoterpene phenolic compound with antimicrobial, antioxidant, anti-inflammatory, anti-cancer, antipyretic, and analgesic properties (Asadi et al., 2023). Therefore, carvacrol has received significant attention from researchers for food applications, notably due to its antibacterial and antibiofilm activities against a broad spectrum of food pathogenic and spoilage strains (Hyldgaard et al., 2012; Hajibonabi et al., 2023). These effects are attributed to the ability of carvacrol to impact the structural and functional characteristics of cytoplasmic membranes. It can expand and destabilize the outer membrane of bacterial strains, thereby increasing their fluidity and cytoplasmic permeability (Lambert et al., 2001; Burt, 2004; Chouhan et al., 2017).

Moreover, in the studies by Wijesundara et al. (2022), it was found that carvacrol induced the deactivation of extracellular polymeric substances (EPS), which were responsible for protecting the bacterial cell against toxic substances and ensuring its strength. Fang et al. (2019) and Luna et al. (2022) determined that the MIC values for carvacrol against *Pseudomonas fluorescens* ATCC 13525 and *E. coli* ATCC 25922 were 0.5 and 0.225 mg/ml, respectively, while Churklam et al. (2020) investigated carvacrol antibacterial activity against *Listeria monocytogenes* strains, including food isolates, with an MIC value of 250 μg/ml. Despite the significant antimicrobial properties of EOs and their compounds, they are not widely used as antimicrobials in the food industry because of their volatility, intense aromas, low solubility, and high susceptibility to oxidation (Hyldgaard et al., 2012; Bakry et al., 2016). Different techniques (e.g., encapsulation, spay drying, and solid lipid nanoparticles) can be used to counterbalance the limitations, ameliorating their biological activity and allowing a controlled release (Ribeiro-Santos et al., 2017; Cacciatore et al., 2022; Mo et al., 2022; Mukurumbira et al., 2022). A feasible approach to reducing this natural compound concentration could be the combination with other natural solutions. Lactic acid bacteria (LAB) represent one of the promising possibilities for the natural control of biofilms that are composed of high antimicrobial resistance and associated risks for foodborne disease spread (Turgis et al., 2012; Esposito and Turku, 2023). LAB can naturally produce antimicrobial compounds, such as organic acids (mainly, lactic acid and acetic acid), diacetyl, hydrogen peroxide, and bacteriocins such as nisin and natamycin, generally recognized as safe (GRAS) (Raman et al., 2022). Organic acid syntheses, such as lactic acid and acetic acid, are mainly responsible for their antagonistic activity against pathogens by acidifying intracellular pH and generating an unfavorable local microenvironment for pathogenic bacteria (Vieco-Saiz et al., 2019; Simons et al., 2020; Martín et al., 2022). In addition, due to its hydrophobic characteristic, the undissolved form of lactic acid and acetic acid enters the cell by passing through the cell membrane, causing alteration, death, and metabolic functions of pathogenic microorganisms (Surendran Nair et al., 2017; Sharma et al., 2022; Mgomi et al., 2023). It has been demonstrated that concentrations of 0.5% (v/v) lactic acid could completely disrupt the growth of pathogenic microorganisms, such as *E. coli, L. monocytogenes*, or *Salmonella* spp. (Wang et al., 2015). The reduction in pH due to the synthesis of LAB organic acids promotes the hydrophobicity of carvacrol, allowing it to explain the bactericidal activity (de Carvalho et al., 2018; Di Gregorio et al., 2022). However, few studies have focused on the synergistic effect of lactic acid bacteria and EOs or their components, showing that the combined treatment may have better antimicrobial activity than single ones and lower concentrations could also be utilized to minimize unwanted side effects (Govaris et al., 2010; Sharma et al., 2022; Esposito and Turku, 2023). Moreover, studies investigating the possibility of using a combination of a pre-formed LAB biofilm monolayer with sub-inhibitory concentrations of natural compounds to enhance whole antimicrobial action are very limited. The synergistic action of LAB monolayer biofilm and carvacrol can be investigated for prospective encapsulation in functional packaging, enabling biofilm formation inhibition and specific health and food biopreservation advantages (Hellebois et al., 2020; Ajlouni et al., 2021). Furthermore, an important aspect to highlight is that the development and application of highly effective natural anti-biofilm treatments for biofilm-related issues in food products and packaging are crucial, given the increasing microbial resistance to antibiotics (Burt, 2004; Asma et al., 2022). This is particularly noteworthy as it has been demonstrated that carvacrol can inhibit microbial growth and biofilm formation without promoting antimicrobial resistance (da Silva et al., 2023). Innovative approaches to assess the culturability and viability of food pathogens in complex matrices are needed to optimize the protocol used for food production and evaluate the effectiveness of preservation treatments (Fleischmann et al., 2021; Rubbens and Props, 2021). It is well-known that using traditional, culture-based microbiological approaches can lead to an overestimation of treatment efficacy (Di Gregorio et al., 2022). Cultivable methods coupled with FCM represent an important strategy for the study of antimicrobial treatment efficacy on both sessile and planktonic cells, providing a deeper understanding of the physiological state of microbial cells. In the present study, we aimed to exploit the *in vitro* bactericidal effect of carvacrol (100 and 250 ppm) enhanced by a pre-formed biofilm monolayer of *Lactiplantibacillus plantarum* against collection strains of foodborne pathogens (*E. coli* and *L. monocytogenes*) and spoilage microorganisms (*P. fluorescens*). The effect of LAB monolayer biofilm and carvacrol was evaluated by using a culture-based approach coupled with flow cytometry (FCM) for monitoring both cultivability and viability of the planktonic and sessile cells. Evaluating the antimicrobial action of bioactive compounds and microbial biofilm's inactivation and degradation activity is essential for developing innovative packaging.

## 2 Materials and methods

### 2.1 Bacterial strains and inoculum preparation

This study used four biofilm-forming strains, i.e., the foodborne pathogens *E. coli* ATCC 25922 and *L. monocytogenes* 56 LY, the food spoilage *P. fluorescens* ATCC 13525, and the probiotic microorganism *L. plantarum* subsp. *plantarum* DSM 20174. A loop of each microbial strain was picked up from glycerol stocks stored at −80°C and grown in 5 ml of culture medium in static condition as follows: *E. coli* and *L. monocytogenes* were grown overnight (o/n) at 37°C in brain heart infusion (BHI) broth (Merck KGaA, Darmstadt, Germany); *P. fluorescens* was grown o/n at 28°C in tryptone soy broth (TSB) (Merck KGaA, Darmstadt, Germany) at 180 rpm, and *L. plantarum* was grown o/n at 30°C in de Man, Rogosa, and Sharpe (MRS) broth (Merck KGaA, Darmstadt, Germany). Then, an aliquot of 1 ml was transferred to 9 ml of fresh medium. After o/n incubation, 1 ml was transferred to 30 ml of fresh sterile medium. Starting from the refreshed culture, the initial concentration of 6 Log CFU/ml (Colony Forming Unit: CFU) for each microorganism was applied for subsequent analysis (Di Gregorio et al., 2022).

### 2.2 Carvacrol solution

Carvacrol, with a purity of ≥98.5%, was purchased from Sigma–Aldrich (St. Louis, MO, United States, ref. W224511) and dissolved in ethanol (absolute, ≥99.8%) to reduce the hydrophobic characteristic. From the 100% stock, an intermediate 10% (v/v) was filtered through a 0.2-mm pore-size filter (Millipore, Bedford, MA, USA) to achieve the final tested concentrations of 50, 100, 250, and 500 ppm. Ethanol alone was used as control for each tested condition (Arioli et al., 2019).

### 2.3 Experimental workflow

The effect of carvacrol, at the concentrations of 50, 100, 250, and 500 ppm, was tested against *L. plantarum* to estimate the probiotic tolerance vs. its planktonic and sessile fraction. Then, the antimicrobial effect of carvacrol (50, 100, 250, and 500 ppm) was assessed against planktonic and biofilm fractions of *E. coli, P. fluorescens*, and *L. monocytogenes*. Subsequently, the combined treatment with a pre-formed biofilm monolayer of the probiotic *L. plantarum* was assessed at the carvacrol concentrations of 100 and 250 ppm. Planktonic and sessile fractions were enumerated through culturability and viability analyses for all tested conditions. Culturability was investigated by using the standard plate count method, and the results were elaborated as logarithmic reduction values (Log CFU/ml) (see Section 2.4). Turbidity and crystal violet assays allowed us to evaluate the antimicrobial efficacy on culturable cells expressed as the percentage of inhibition and calculated as described by Bazargani and Rohloff (2016). The flow cytometry viability results were expressed as percentage values of the internal cell subpopulations, which were differentiated by membrane integrity (see Section 2.6). Three biological replicates from three independent experiments were considered for each tested condition.

### 2.4 Biofilm formation

An aliquot of bacterial suspension (0.05 ml) at the concentration of 6 Log cells/ml was added to sterile tubes containing 4.9 ml of sterile growth medium (BHI for *E. coli* and *L. monocytogenes*, TSB for *P. fluorescens*, and MRS for *L. plantarum*) and 0.05 ml of carvacrol solution at the final concentrations of 50, 100, 250, and 500 ppm. The experiment was performed in triplicate with a rapid microplate methodology using 96-well plates (Corning Costar 96-well, flat-bottom microplate) (Bragonzi et al., 2012). The four plates, one for each microorganism, were incubated overnight in static condition at 37°C for *E. coli* and *L. monocytogenes*, 28°C for *P. fluorescens*, and 30°C for *L. plantarum*. Uninoculated controls were present in each plate. After 24 h of incubation, the planktonic cell fractions were transferred to new microtiter plates (see below) while the attached cells were rinsed three times with 0.2 ml of phosphate-buffered saline (PBS, pH 7.4) to remove non-adherent and weakly adherent bacteria.

#### 2.4.1 CV assay

The sessile cells were quantified using the crystal violet assay as described in the study by Bragonzi et al. (2012). In brief, plates were air-dried for 30 min at room temperature, and biofilms were stained with 0.2 ml of 1% (w/v) solution of crystal violet (CV) in water. After 20 min of staining at room temperature, the samples were washed thrice by dipping each sample in 200 μl of sterile PBS. The bound dye was dissolved by adding 0.2 ml of 95% (v/v) ethanol to de-stain the samples. The quantitative analysis of biofilm production was performed by measuring the absorbance at 595 nm with the Promega™ GloMax^®^ automated reader. To compensate for background absorbance, OD readings from sterile medium, dye, and ethanol were averaged and subtracted from all test values.

#### 2.4.2 Planktonic fraction

To correlate biofilm formation with the growth of planktonic cells in each well, the planktonic cell fractions, previously transferred to new microtiter plates as described above, were quantified by plating 0.1 ml of 10-fold bacterial suspension serial dilutions (NaCl 0.9% w/v) on BHI (for the enumeration of *E. coli* and *L. monocytogenes*), TSB (for the enumeration of *P. fluorescens*), and MRS (for the enumeration of *L. plantarum*) agar plates, following the procedure reported by Bragonzi et al. (2012). After 24 h of incubation at 37, 28, and 30°C, for *E. coli, L. monocytogenes, P. fluorescens*, and *L. plantarum*, respectively, colonies were counted. For the turbidity assay, microtiter plates were measured (OD 595 nm) by using a microplate automated reader (Promega™ GloMax^®^). The viability of planktonic cells was investigated through a flow cytometry approach (see Section 2.6).

#### 2.4.3 Sessile fraction

To enumerate the sessile (adherent) cells of tested bacteria, the wells, after removing the planktonic fraction, were rinsed three times with 200 ml of PBS to remove non-adherent and weakly adherent bacteria. Then, the biofilm was removed by scraping the surface of each well with 1 ml of PBS, and the recovered cells were suspended by vortexing for 30 s. The number of sessile cells was determined by plating appropriate dilutions of biofilm samples on agar media, as described above. The viability of sessile cells was investigated through a flow cytometry approach (see Section 2.6).

### 2.5 *L. plantarum* pre-formed biofilm

The combined effect of carvacrol at concentrations of 100 and 250 ppm, with a pre-formed biofilm of *L. plantarum*, was investigated on *E. coli, L. monocytogenes*, and *P. fluorescens*. First, an aliquot of 0.2 ml of untreated *L. plantarum* (6 Log CFU/ml) was inoculated into 96-well plates, one for each microorganism, to avoid contaminations. After 24 h of incubation at 30°C, the *L. plantarum* planktonic fraction was removed, and each well was rinsed three times with 0.2 ml PBS to take off non-adherent and weakly adherent cells. In total, 0.2 ml of each bacterial strain (*E. coli, L. monocytogenes*, and *P. fluorescens*) at the concentration of 6 Log CFU/ml was added to the *L. plantarum* pre-formed biofilm wells. The experimental conditions for each microorganism were set up as follows:

i. *L. plantarum* preformed biofilm + microorganism (*L.p*.).
ii. *L. plantarum* preformed biofilm + carvacrol 100 ppm + microorganism (*L.p*.+100 ppm).
iii. *L. plantarum* preformed biofilm + carvacrol 250 ppm + microorganism (*L.p*.+250 ppm).

As a control, 0 ppm samples and the single treatments with carvacrol 100 ppm and carvacrol 250 ppm for each strain were used. Autoclaved bacterial suspension and uninoculated fresh sterile medium were added to each 96-well plate as a negative control. After 24 h of incubation, planktonic and sessile cells were analyzed to investigate the combined effect of *L. plantarum* pre-formed biofilm with carvacrol (see Section 2.4). Specifically, for the standard plate count method, samples obtained from the *L. plantarum* pre-formed biofilm test were also plated on MRS agar to subtract the CFU of *L. plantarum* from the whole colonies observed. The viability of cells was assessed by flow cytometry as described in Section 2.6.

### 2.6 Flow cytometry assay

*E. coli, L. monocytogenes, P. fluorescens*, and *L. plantarum* samples were diluted 100-fold in PBS, and a double staining SYTO24 (1x, ThermoFisher, USA)/Propidium Iodide (PI) (10 μg/ml) was performed. The stained cell suspensions were distributed in triplicate in 96-well plates and incubated for 15 min at 37°C before testing. Each sample utilized microspheres of 2.5 μm in diameter (Alignflow™ for Blue Lasers, Thermo Fisher Scientific Life Science Solutions, Milan, Italy) as internal reference standards. CytoFLEX S flow analyzer (Beckman Coulter, Flow Cytometry, Milan, Italy) was used by applying FSC 10,000 threshold settings. Each sample acquired 50,000 events at a slow flow rate (10 μl/min). Blue laser source (est. 488 nm) was used, and bandpass filters BP525/40 nm and BP675/50 nm were selected to collect fluorescence emission of SYTO24 and PI, respectively. The double staining method allowed us to distinguish three populations based on the three states of cell damage, namely, viable, injured, and dead cells, identified on a SYTO24 vs. PI dot plot according to the different ratio of green/red fluorescence signals from the positive and negative (autoclaved cells) control (Di Gregorio et al., 2022). The signals were measured as Total Fluorescent Units (TFUs), including all stained cells emitting fluorescence and embracing viable, dead, and injured cells. The ability to discriminate viable cells from damaged and dead cells allowed the numbering of viable cells as Active Fluorescent Units (AFU: total cell number minus damaged and dead ones) (Foglia et al., 2020; Di Gregorio et al., 2022). The parameters were acquired with a logarithmic scale and analyzed using CytExpert software v. 2.3 (Beckman Coulter Flow Cytometry, Milan, Italy). Cellular internal distribution results were expressed as percentage of alive, injured, and dead cells computed from the total FCM recorded events (50,000 events), excluding the background and standard bead signals. Each percentage was obtained as the average of three replicates from three independent experiments, and the standard deviation was always <5%.

### 2.7 Statistical analysis

The statistical analysis was performed by using ^©^GraphPad Prism software (version 9.5.1). Data are presented as mean ± standard deviations (SD) based on triplicates from at least three independent experiments. Data were compared using two-way ANOVA in Dunnett's multiple comparison tests with Tukey's pairwise test at *p* < 0.05 considered statistically significant (95% confidence interval).

## 3 Results

### 3.1 Carvacrol effect on *L. plantarum* DSM 20174 probiotic strain

#### 3.1.1 Planktonic cells

**Figure 1:**
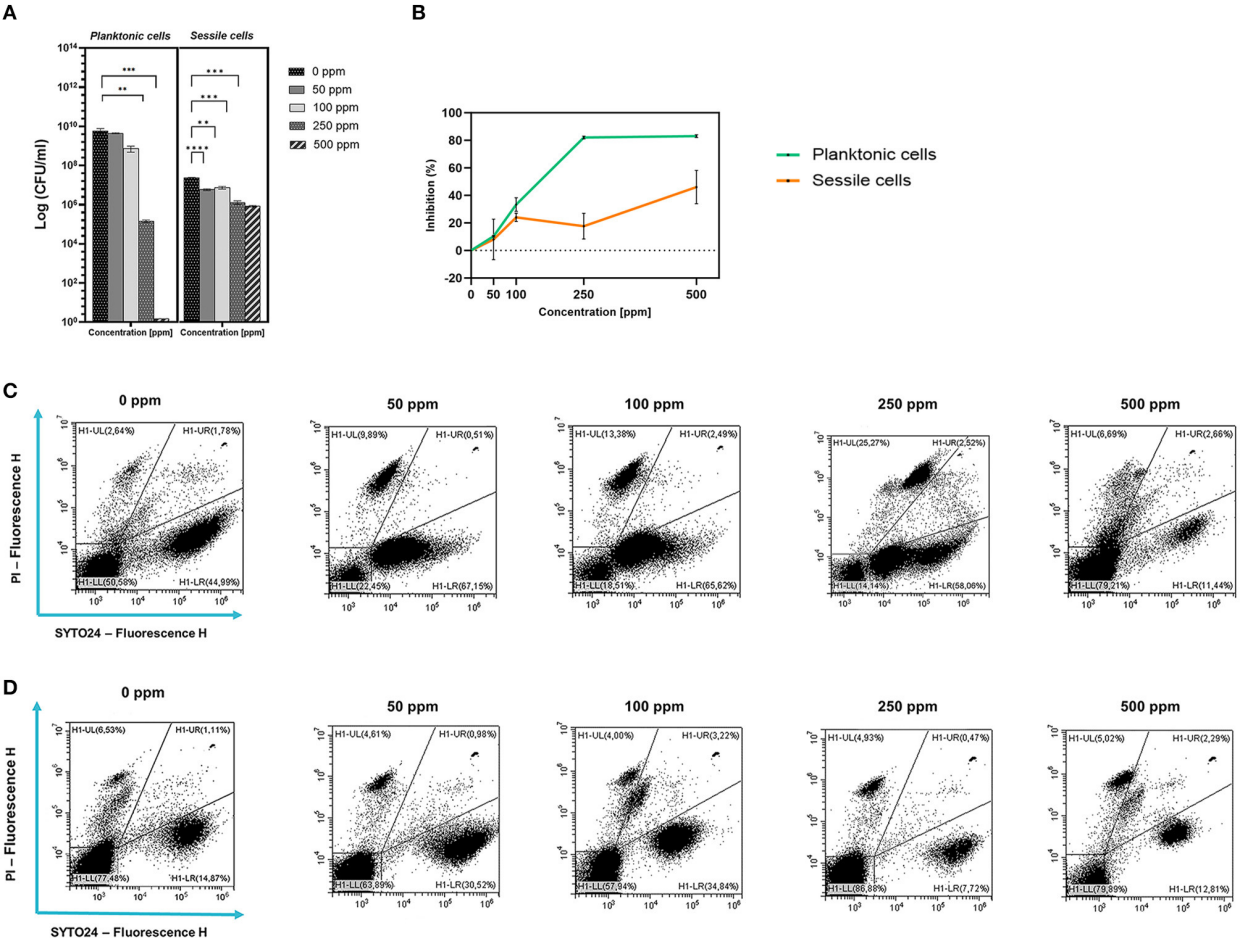
Effect of carvacrol against planktonic and sessile cells of *L. plantarum DSM 20174* grown in MRS broth and plated on MRS agar plate. Histograms indicate the Log CFU/ml values after antimicrobial treatment with increasing concentration of carvacrol (50, 100, 250, and 500 ppm) compared to control (0 ppm) after 24 h of incubation. (*) *p* < 0.05, (**) *p* < 0.01, (***) *p* < 0.001, and (****) *p* < 0.0001 **(A)**. Carvacrol antimicrobial treatment efficacy on *L. plantarum* DSM 20174 planktonic (turbidimetric analysis, OD 595 nm) and sessile cells (CV assay) grown in MRS broth. Graphs indicate the inhibition growth percentage values after antimicrobial treatments (carvacrol 50, 100, 250, and 500 ppm) compared with control (0 ppm) after 24 h of incubation **(B)**. Data **(A, B)** are presented as mean ± standard deviations (SD) based on triplicates from three independent experiments. FCM double-staining (SYTO24 and PI) dot plot of *L. plantarum* DSM 20174 planktonic **(C)** and sessile cells **(D)** grown in MRS broth in the presence of carvacrol (50, 100, 250, and 500 ppm) and diluted in PBS (pH 7.4). The results were compared with control (0 ppm) after 24 h of incubation. H1-LL, unstained debris; H1-LR, intact cells/viable cells (SYTO24 positive); H1-UR, injured cell population; H1-UL, permeabilized/dead cells (PI positive).

Plate-count results showed a growth reduction that was proportional to the increasing concentration of carvacrol added. Significant results after carvacrol 250 and 500 ppm treatments were observed (Figure 1A). The highest carvacrol concentration (500 ppm) promoted a total loss of cultivability, while a logarithmic reduction of 4.62 was calculated after the application of carvacrol 250 ppm. However, the presence of carvacrol at concentrations of 50 and 100 ppm did not result in significant reductions in probiotic growth. A turbidimetric analysis also showed greater efficacy of 250 and 500 ppm carvacrol treatments. A higher inhibition percentage of 83.7% was observed when carvacrol at 500 ppm was used, confirming the loss of culturability detected using the culture-based methods (Figure 1B). Nevertheless, the FCM results showed that after carvacrol 500 ppm treatment, 42% of the cell population was still viable with no further ability to replicate on culture media, suggesting the transition of cells into the VBNC state treatment-induced (15% injured and 43% dead cells) (Figure 1C). Furthermore, FCM showed that after carvacrol 250 ppm treatment, the cells were mostly viable (63% alive, 16% damaged, and 21% dead).

#### 3.1.2 Sessile cells

Despite the loss of planktonic cell culturability, *L. plantarum* sessile fraction was less sensitive to carvacrol antimicrobial treatment (1.27 and 1.46 log reduction for 250 and 500 ppm, respectively). However, the reduction values were significant at 50 ppm concentration. A different behavior was observed when samples were analyzed using the CV assay (Figure 1). The highest percentage of inhibition was found after carvacrol 500 ppm treatment (46%), while carvacrol 250 ppm treatment resulted in 17.7%. An FCM analysis confirmed the resistance of *L. plantarum* sessile cells, showing a higher degree of damage at the carvacrol 100 and 500 ppm concentrations (29 and 21%, respectively), and a high percentage of alive cells was maintained at all tested concentrations (72, 53, 61, and 64% for 50, 100, 250, and 500 ppm, respectively).

### 3.2 Carvacrol effect on *E. coli, P. fluorescens*, and *L. monocytogenes* planktonic cells

**Figure 2:**
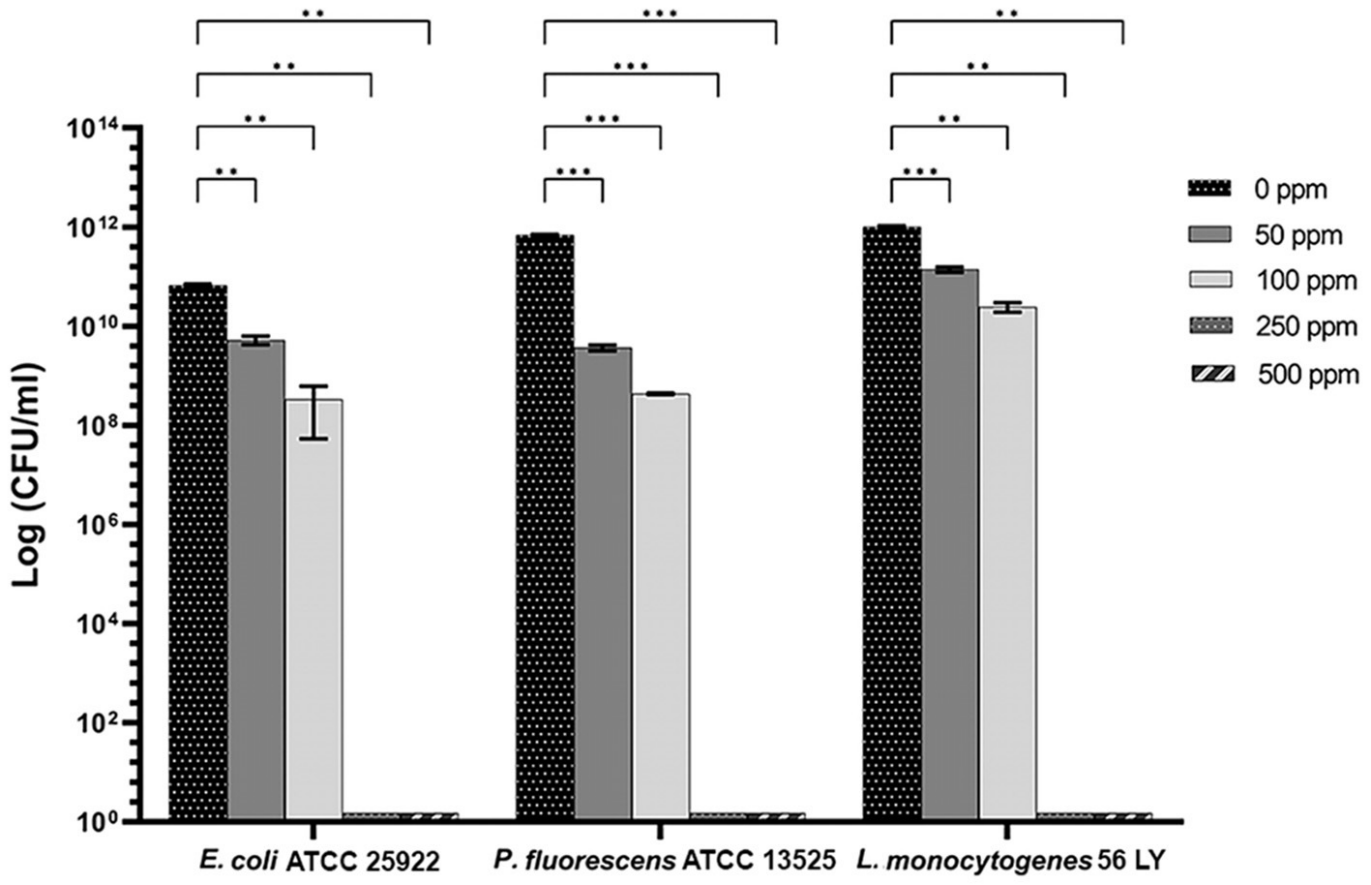
Effect of carvacrol against planktonic cells of *E. coli* ATCC 25922, *P. fluorescens* ATCC 13525, and *L. monocytogenes* 56 LY, grown in BHI broth (*E. coli* and *L. monocytogenes*) and TSB broth (*P. fluorescens*) and plated on BHI agar (*E. coli* and *L. monocytogenes*) and TSA agar (*P. fluorescens*). Histograms indicate the Log CFU/ml values after antimicrobial treatment with increasing concentrations of carvacrol (50, 100, 250, and 500 ppm) compared with control (0 ppm) after 24 h of incubation. Data are presented as mean ± standard deviations (SD) based on triplicates from three independent experiments. (*) *p* < 0.05, (**) *p* < 0.01, (***) *p* < 0.001, and (****) *p* < 0.0001.

**Figure 3:**
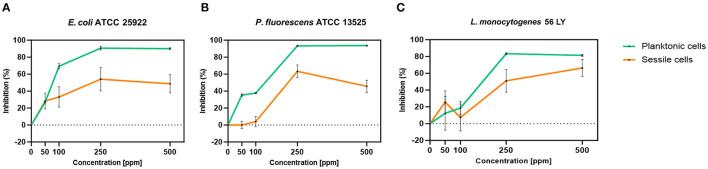
Carvacrol antimicrobial treatment efficacy on *E. coli* ATCC 25922 **(A)**, *P. fluorescens* ATCC 13525 **(B)**, and *L. monocytogenes* 56 LY **(C)** planktonic (turbidimetric analysis, OD 595 nm) and sessile cells (CV assay), grown in BHI broth (*E. coli* and *L. monocytogenes*) and TSB broth (*P. fluorescens*). Graphs indicate the inhibition growth percentage values after antimicrobial treatments (carvacrol 50, 100, 250, and 500 ppm) compared with control (0 ppm), after 24 h of incubation. Data are presented as mean ± standard deviations (SD) based on triplicates from three independent experiments.

**Figure 4:**
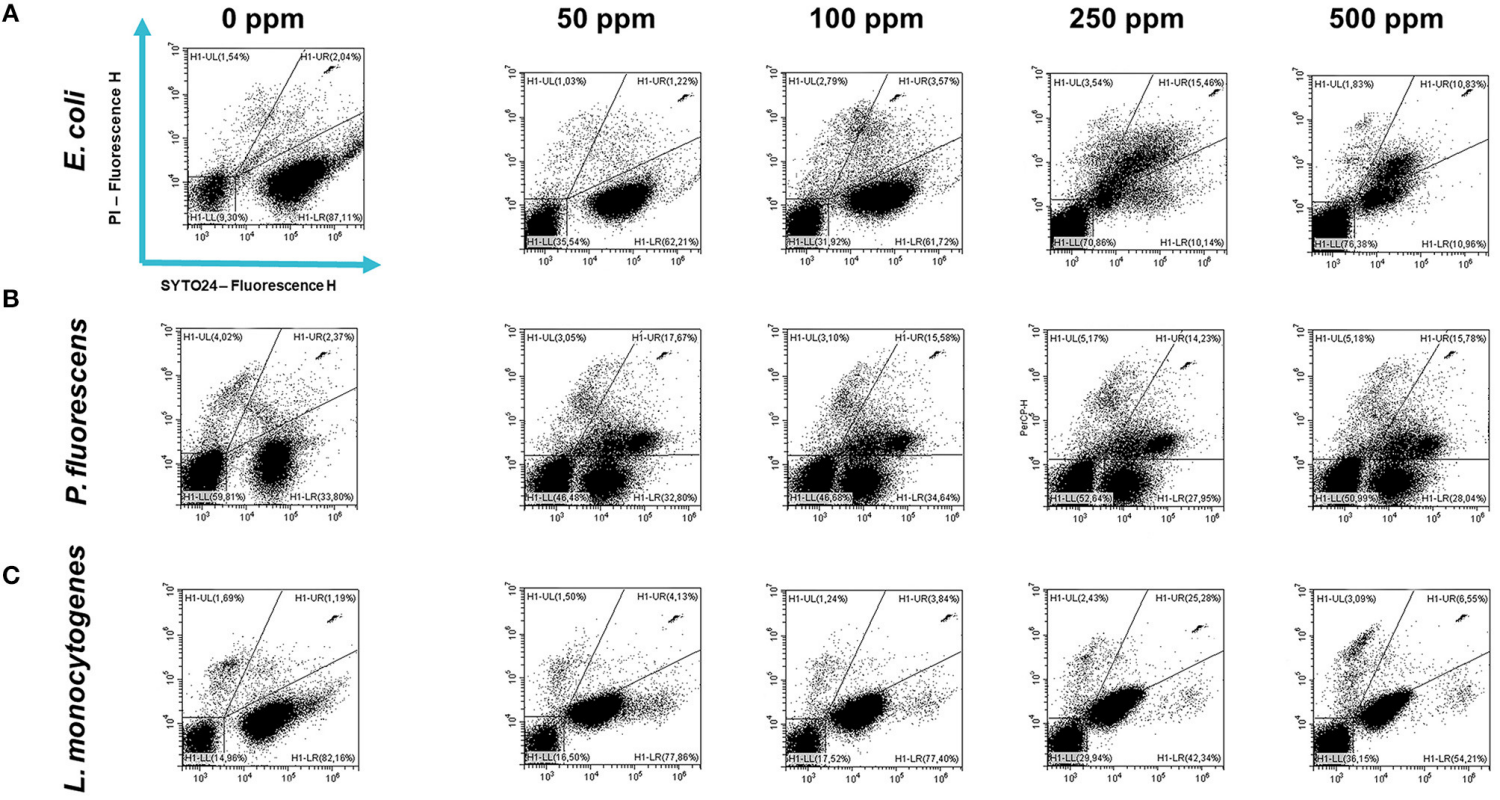
Double-staining dot plot of *E. coli* ATCC 25922 **(A)**, *P. fluorescens* ATCC 13525 **(B)**, and *L. monocytogenes* 56 LY **(C)** planktonic cells grown in BHI broth (*E. coli* and *L. monocytogenes*) and TSB broth (*P. fluorescens*) in the presence of carvacrol (50, 100, 250, and 500 ppm) and diluted in PBS (pH 7.4). The results were compared with 0 ppm (control) after 24 h of incubation. Cells were stained with SYTO24 and PI simultaneously. H1-LL, unstained debris; H1-LR, intact cells/viable cells (SYTO24); H1-UR, injured cell population; H1-UL, permeabilized/dead cells (PI).

Carvacrol treatment had a significant antimicrobial action on *E. coli, P. fluorescens*, and *L. monocytogenes* planktonic cells for all tested concentrations (*p* < 0.05) compared with the control (Figure 2). Overall, 250 and 500 ppm concentrations promoted a total loss of culturability in each strain. A high logarithmic reduction in *P. fluorescens* cells compared with the control was observed at 2.27 and 3.20 when 50 and 100 ppm concentrations of carvacrol were applied. For the same concentrations, the *E. coli* cell growth was reduced to 1.10 and 2.29 and to 0.86 and 1.61 in *L. monocytogenes*, respectively. According to the plate-count results, after the addition of carvacrol 250 and 500 ppm, more than 90% of inhibition in *E. coli* e *P. fluorescens* was calculated by turbidimetric analysis, and 82 and 78% of inhibition were estimated in *L. monocytogenes* (Figures 3A–C). In all tested strains, 250 ppm was the minimum concentration which was able to inhibit at least 80% of the cell population. In *P. fluorescens*, inhibition of 37% was observed already after 50 ppm treatment, compared with 25 and 12% in *E. coli* and *L. monocytogenes* for the same concentration, respectively. *L. monocytogenes* was shown to be the least susceptible strain to carvacrol antimicrobial action (Figures 3A–C). The dot plot obtained from FCM analysis showed different microorganisms' responses to the treatments (Figure 4): Planktonic cells of *E. coli* exhibited a proportional damage degree up to the 250 ppm carvacrol concentration, while after 500 ppm treatment, part of the cells recovered viability resulting in 46% viable cells. Therefore, the FCM analysis suggested that the cells were unculturable but still viable (VBNC) (Figure 4A). Samples treated with 50 ppm maintained a viable cell fraction of 84% compared with 87% alive cells of the control. Using the 100 ppm concentration, injured and dead cells increased to 12 and 23%, respectively, while at 250 ppm concentration, 61% were damaged and only 27 and 12% were viable and dead, respectively. However, in *P. fluorescens* and *L. monocytogenes*, the percentage of viable cells remained almost unchanged after treatments: in *P. fluorescens*, viable cells were 52, 48, 51, and 56% for carvacrol at 50, 100, 250, and 500 ppm concentrations, respectively, while in *L. monocytogenes*, they were 71, 68, 59, and 43% for the same increasing concentrations of carvacrol, respectively (Figures 4B, C).

### 3.3 Carvacrol effect on *E. coli, P. fluorescens*, and *L. monocytogenes* sessile cells

**Figure 5:**
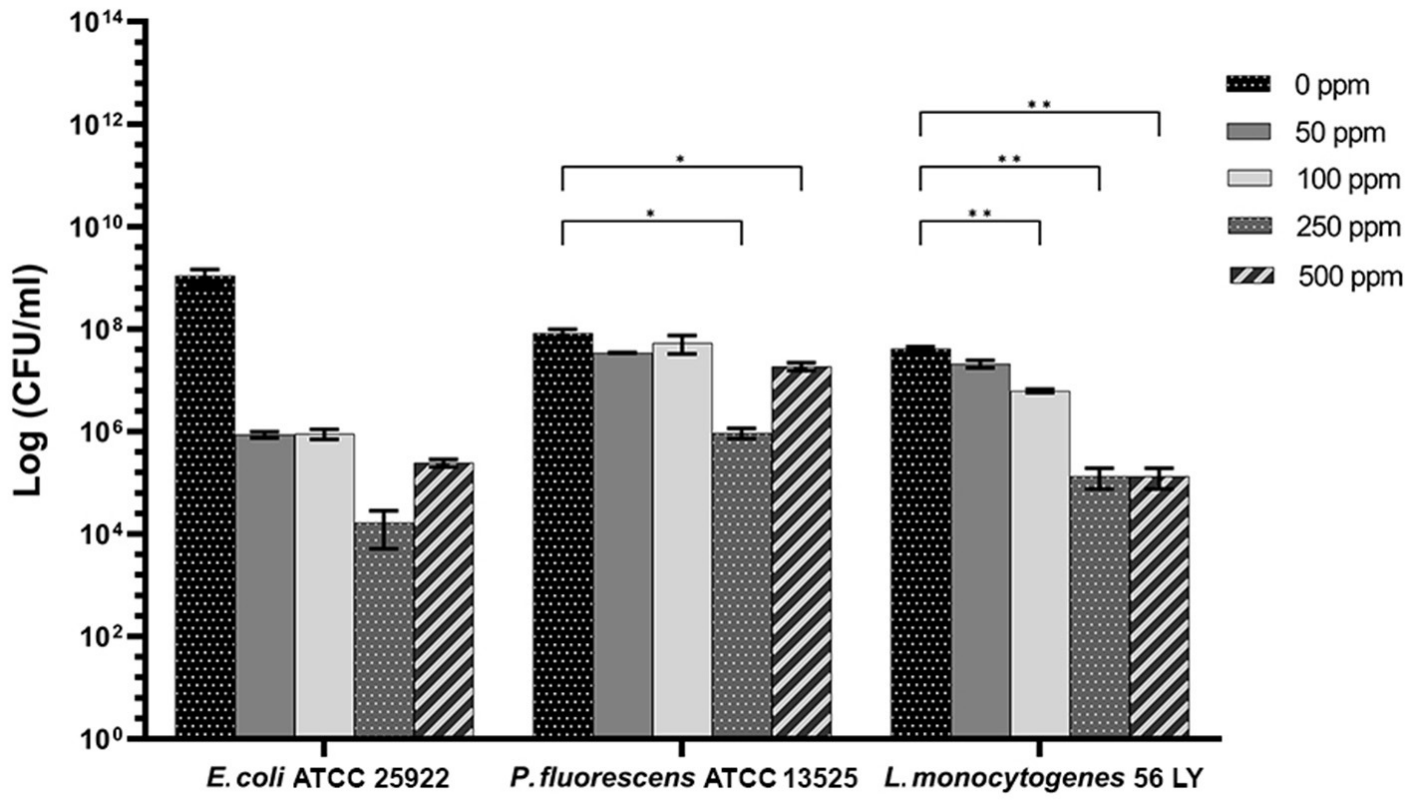
Effect of carvacrol against sessile cells of *E. coli* ATCC 25922, *P. fluorescens* ATCC 13525, and *L. monocytogenes* 56 LY grown in BHI broth (*E. coli* and *L. monocytogenes*) and TSB broth (*P. fluorescens*) and plated on BHI agar (*E. coli* and *L. monocytogenes*) and TSA agar (*P. fluorescens*). Histograms indicate the Log CFU/ml values after antimicrobial treatment with increasing concentrations of carvacrol (50, 100, 250, and 500 ppm) compared with control (0 ppm) after 24 h of incubation. Data are presented as mean ± standard deviations (SD) based on triplicates from three independent experiments. (*) *p* < 0.05, (**) *p* < 0.01, (***) *p* < 0.001, and (****) *p* < 0.0001.

**Figure 6:**
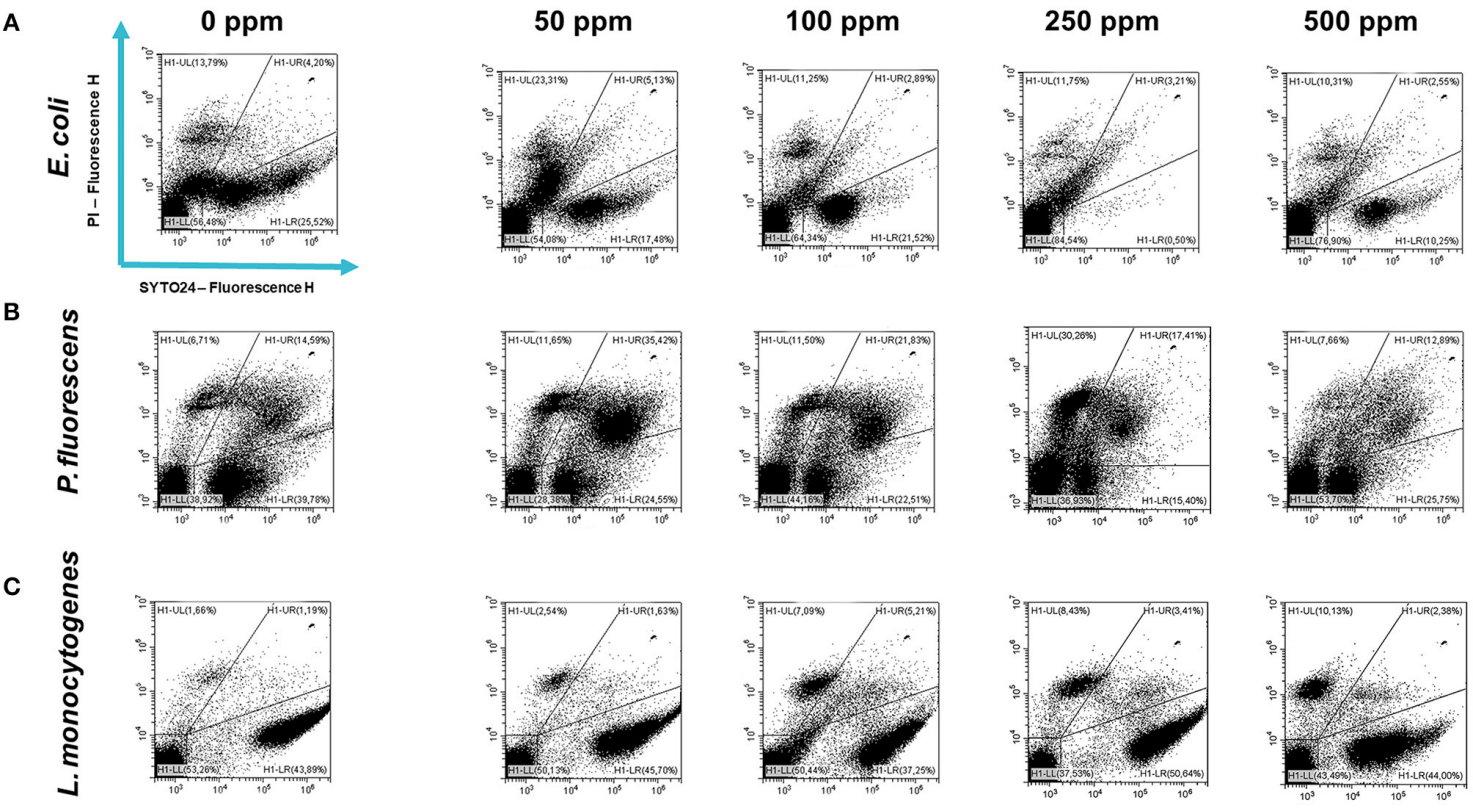
Double-staining dot plot of *E. coli* ATCC 25922 **(A)**, *P. fluorescens* ATCC 13525 **(B)**, and *L. monocytogenes* 56 LY **(C)** sessile cells grown in BHI broth (*E. coli* and *L. monocytogenes*) and TSB broth (*P. fluorescens*) in the presence of carvacrol (50, 100, 250, and 500 ppm) and diluted in PBS (pH 7.4). The results were compared with control (0 ppm) after 24 h of incubation. Cells were stained with SYTO24 and PI simultaneously. H1-LL, unstained debris; H1-LR, intact cells/viable cells (SYTO24); H1-UR, injured cell population; H1-UL, permeabilized/dead cells (PI).

Carvacrol antimicrobial action had a significant effect on *P. fluorescens* sessile fraction when 250 and 500 ppm concentrations were applied (1.95 and 0.65 log reduction, respectively) (*p* < 0.05) and on *L. monocytogenes* after 100, 250, and 500 ppm treatments (0.82, 2.49, and 2.49, respectively) (*p* < 0.05). For *E. coli*, a logarithmic reduction of 3.10, 3.09, 4.82, and 3.66 after carvacrol 50, 100, 250, and 500 ppm treatments was found, respectively (*p* > 0.05) (Figure 5). Based on the culture-based results, carvacrol 250 ppm proved to be the most effective minimum concentration in all tested strains, whereas increasing to 500 ppm did not enhance the antimicrobial effect (Figure 5). Unlike the planktonic fraction, no loss of culturability in any strain was observed; indeed, the minimum growth level after treatment was 10^4^ Log CFU/ml (*E. coli*, carvacrol 250 ppm) (Figures 2, 5). The CV test showed a percentage of inhibition of 52 and 63% for *E. coli* and *P. fluorescens*, respectively, while in *L. monocytogenes*, the higher efficacy was obtained after carvacrol 500 ppm (65% reduction compared with 48% inhibition related to carvacrol 250 ppm) (Figure 3C). FCM analysis confirmed a greater carvacrol effect at 250 ppm concentration in *E. coli* e *P. fluorescens*. *E. coli* viability fraction was down to 4% in favor of a highly damaged state, which represented 73% of cells. Nevertheless, after the carvacrol 500 ppm treatment, 36% of cells persisted viable, and 27 and 37% were injured and dead cells, respectively (Figure 6A). Overall, 50 ppm concentration increased the damaged state (33 %) compared with the 100 ppm concentration, where 43% of cells were viable, 21% were injured, and 36% were dead cells. *P. fluorescens* displayed an increase in damaged cells as early as 50 ppm concentration (51% compared with 22% of the control), resulting in a bactericidal effect when the 250 ppm concentration was applied: 23% alive, 31% injured, and 46% dead cells (Figure 6B). The most notable percentages of dead cells were observed at concentrations of 50 and 250 ppm (31 and 46%); indeed, after 100 ppm treatment, a percentage of 42% was still viable. Although 500 ppm was the highest concentration used, this resulted in a high fraction of viable cells after treatment (68%), and only 15 and 17% were damaged and dead, respectively. On the other hand, in *L. monocytogenes*, minor reductions in viable cells were observed (68, 61, 57, and 54% at 50, 100, 250, and 500 ppm, respectively) (Figure 6C).

### 3.4 Pre-formed *L. plantarum* biofilm combined with carvacrol against *E. coli, P. fluorescens*, and *L. monocytogenes*

#### 3.4.1 Planktonic cells

**Figure 7:**
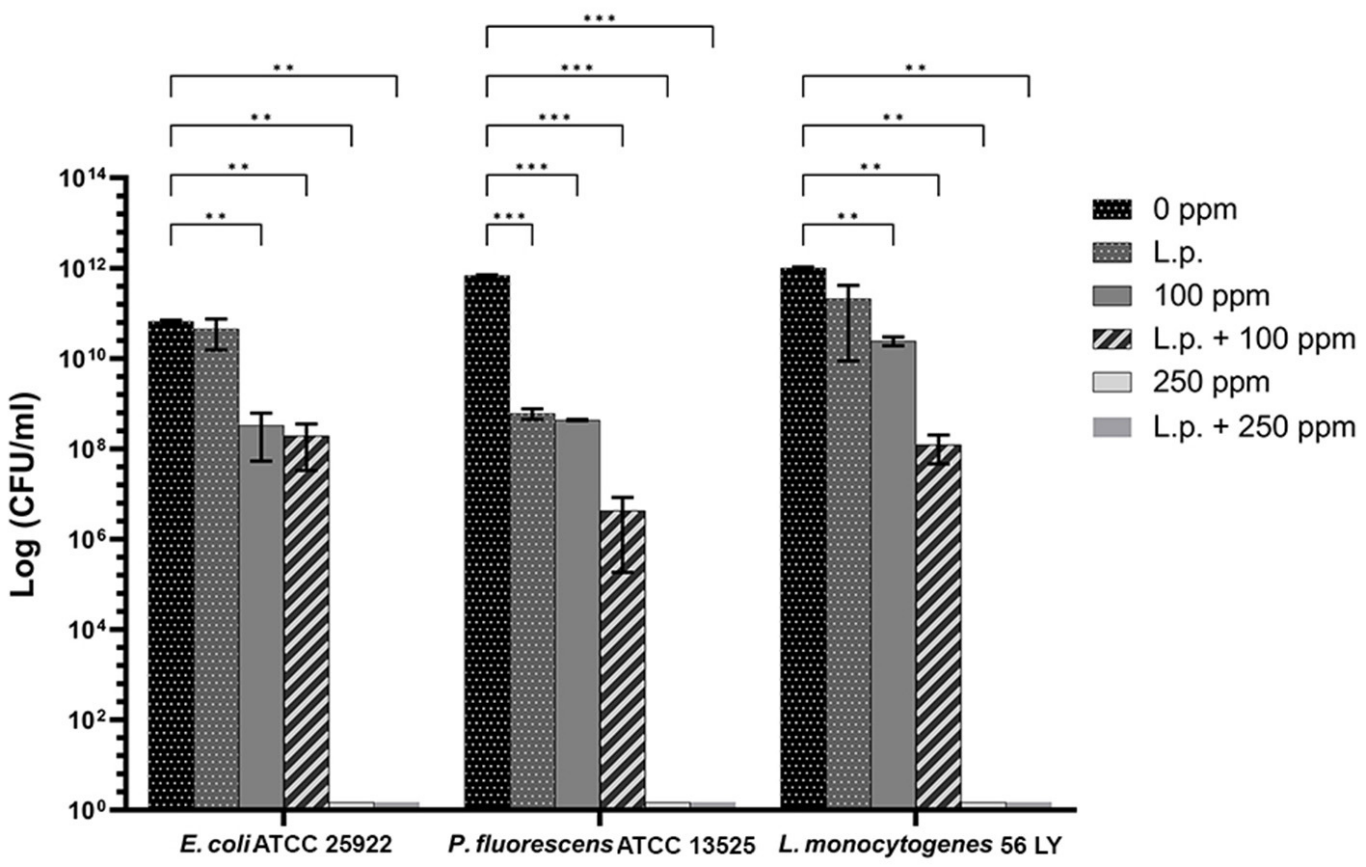
Effect of *L. plantarum* pre-formed biofilm against planktonic cells of *E. coli* ATCC 25922, *P. fluorescens* ATCC 13525, and *L. monocytogenes* 56 LY grown in BHI broth (*E. coli* and *L. monocytogenes*) and TSB broth (*P. fluorescens*), and plated on BHI agar (*E. coli* and *L. monocytogenes*) and TSA agar (*P. fluorescens*). Histograms indicate the Log CFU/mL values after antimicrobial treatments: single treatments with *L. plantarum* pre-formed biofilm (*L.p*.), carvacrol 100 ppm, and carvacrol 250 ppm against each strain, and combined treatments with carvacrol 100 ppm (*L.p*. + 100 ppm) and 250 ppm (*L.p*. + 250 ppm), compared to control (0 ppm) after 24 h of incubation. Data are presented as mean ± standard deviations (SD) based on triplicates from three independent experiments. (*) *p* < 0.05, (**) *p* < 0.01, (***) *p* < 0.001, (****) *p* < 0.0001.

**Figure 8:**
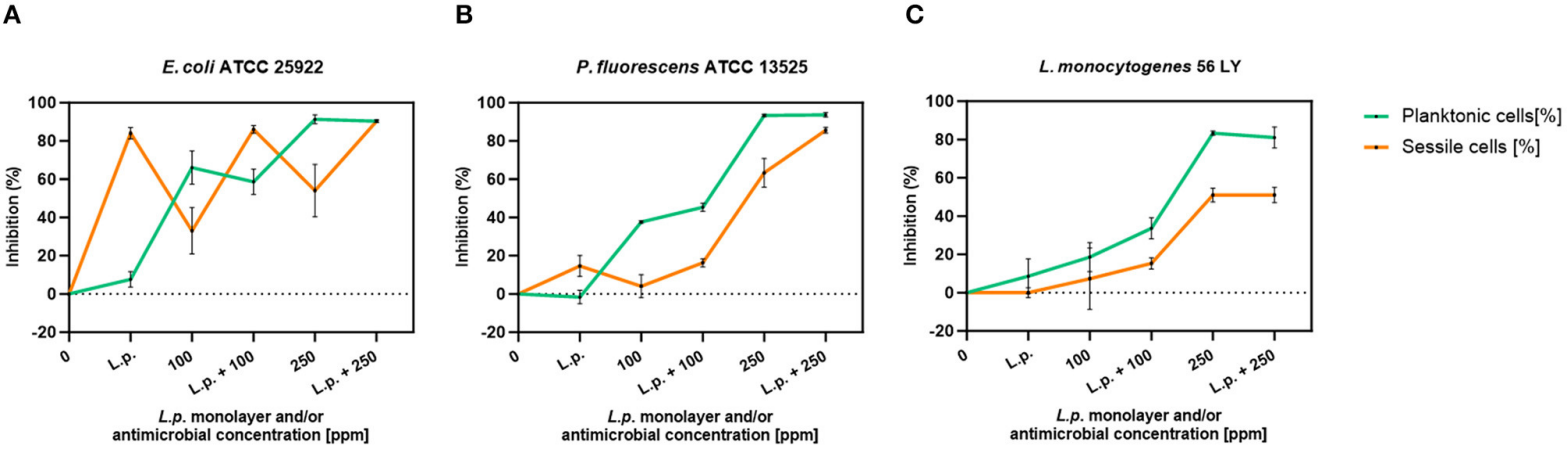
*L. plantarum* pre-formed biofilm efficacy on *E. coli* ATCC 25922 **(A)**, *P. fluorescens* ATCC 13525 **(B)**, and *L. monocytogenes* 56 LY **(C)** planktonic (turbidimetric analysis, OD 595 nm) and sessile cells (CV assay) grown in BHI broth (*E. coli* and *L. monocytogenes*) and TSB broth (*P. fluorescens*). Graphs indicate the inhibition growth percentage values after antimicrobial treatments: single treatments with *L. plantarum* pre-formed biofilm alone (*L.p*.), carvacrol 100 and 250 ppm, and combined treatments with carvacrol 100 ppm (*L.p*. + 100 ppm) and 250 ppm (*L.p*. + 250 ppm), compared with control (0 ppm) after 24 h of incubation. Data are presented as mean ± standard deviations (SD) based on triplicates from three independent experiments.

**Figure 9:**
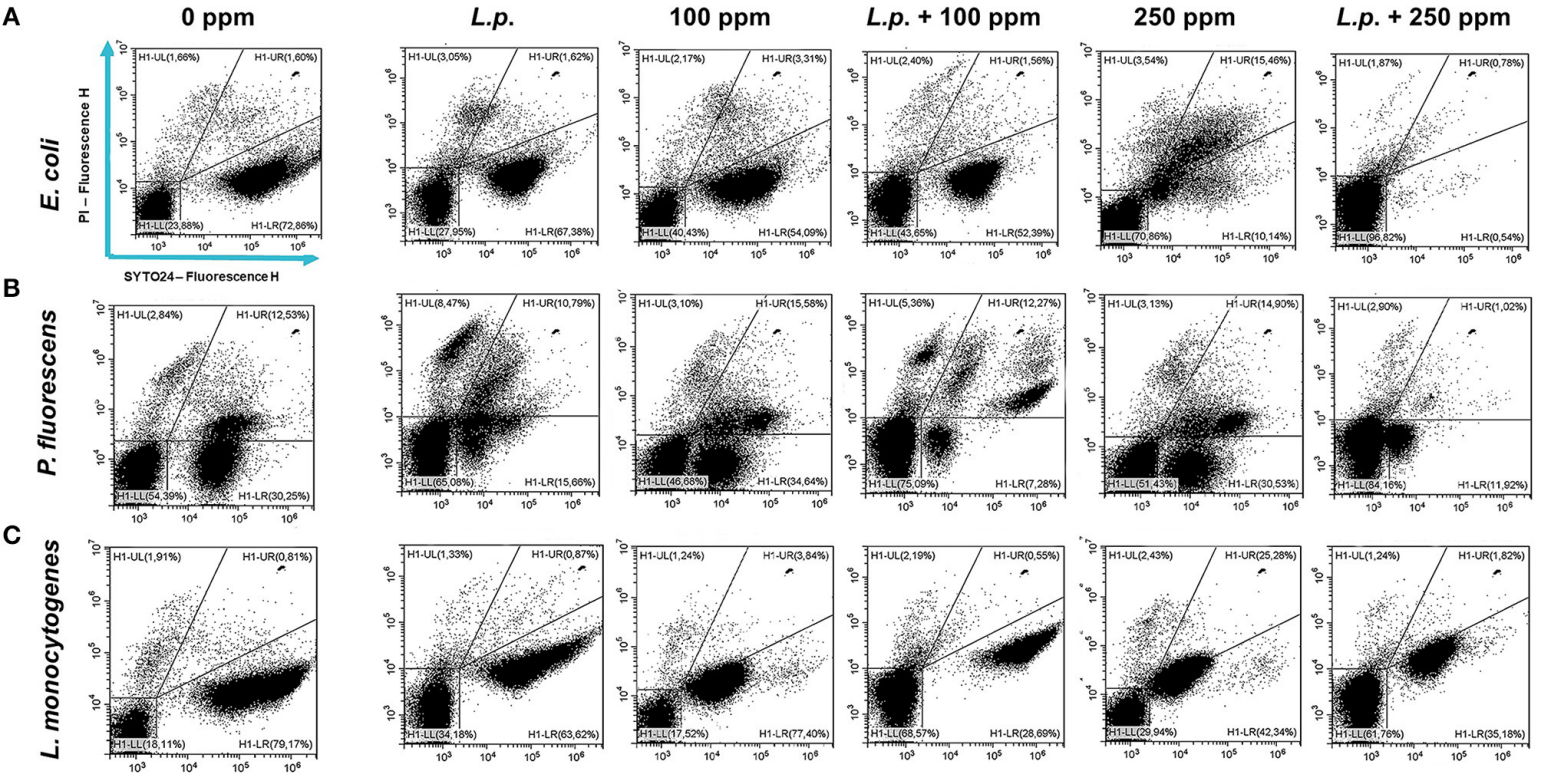
Double-staining dot plot of *E. coli* ATCC 25922 **(A)**, *P. fluorescens* ATCC 13525 **(B)**, and *L. monocytogenes* 56 LY **(C)** planktonic cells grown in the presence of *L. plantarum* pre-formed biofilm and diluted in PBS (pH 7.4). The addition of *L. plantarum* pre-formed biofilm (*L.p*.) to tested strains and combined treatments with carvacrol 100 ppm (*L.p*. + 100 ppm) and 250 ppm (*L.p*. + 250 ppm), compared with control (0 ppm), carvacrol 100 and 250 ppm single treatments, after 24 h of incubation. Cells were stained with SYTO24 and PI simultaneously. H1-LL, unstained debris; H1-LR, intact cells/viable cells (SYTO24); H1-UR, injured cell population; H1-UL, permeabilized/dead cells (PI).

The presence of pre-formed biofilm evaluated by the culture-based methods determined a significant growth reduction in *P. fluorescens* (2.82) (*p* < 0.05) (Figure 7). The combined effect of *L. plantarum* +100 ppm caused a significant reduction in all tested strains (*p* < 0.05) compared with carvacrol treatment alone: *L. plantarum* + 100 ppm promoted a reduction of 2.54 in *E. coli*, 6.22 in *P. fluorescens*, and 3.64 in *L. monocytogenes*. Moreover, the turbidity assay showed a growth inhibition of 58, 43, and 32% in *E. coli, P. fluorescens*, and *L. monocytogenes*, respectively (Figures 8A–C). A total loss of culturability in all samples that were treated with only carvacrol 250 ppm and *L. plantarum* + 250 ppm was observed (Figure 7). The logarithmic reduction detected by plate count was confirmed by the turbidity assay, where more than 80% of inhibition was detected (Figures 7, 8). FCM analysis emphasized the difference between the bactericidal effect of combined *L. plantarum* + 250 ppm treatment and the bacteriostatic action of carvacrol 250 ppm alone (69% damaged cells) against *E. coli* (Figure 9A). The presence of pre-formed biofilm induced a strong cellular impairment, leading to an increase in the background signal caused by the presence of debris, while the combined treatment (*L. plantarum* + 100 ppm) showed no substantial differences from the single one (100 ppm) (Figure 9A). In *P. fluorescens*, the antimicrobial action of single treatment with pre-formed biofilm (*L.p*.) was already evident in terms of cellular damage and death (26 and 44%, respectively) compared with the 100 ppm carvacrol treatment alone (42 and 13%, respectively). Moreover, *P. fluorescens* samples treated with carvacrol 100 ppm showed a reduction in viable fraction from the single (45%) to the combined treatment (26%). The *L. plantarum* + 250 treatment compromised a cellular membrane with a subsequent increase in the background signal, although a viable fraction of 32% persisted but was still lower than the 250 ppm single treatment (48%) (Figure 9B). Finally, the internal population distribution of *L. monocytogenes* was not significantly changed (Figure 9C).

#### 3.4.2 Sessile cells

**Figure 10:**
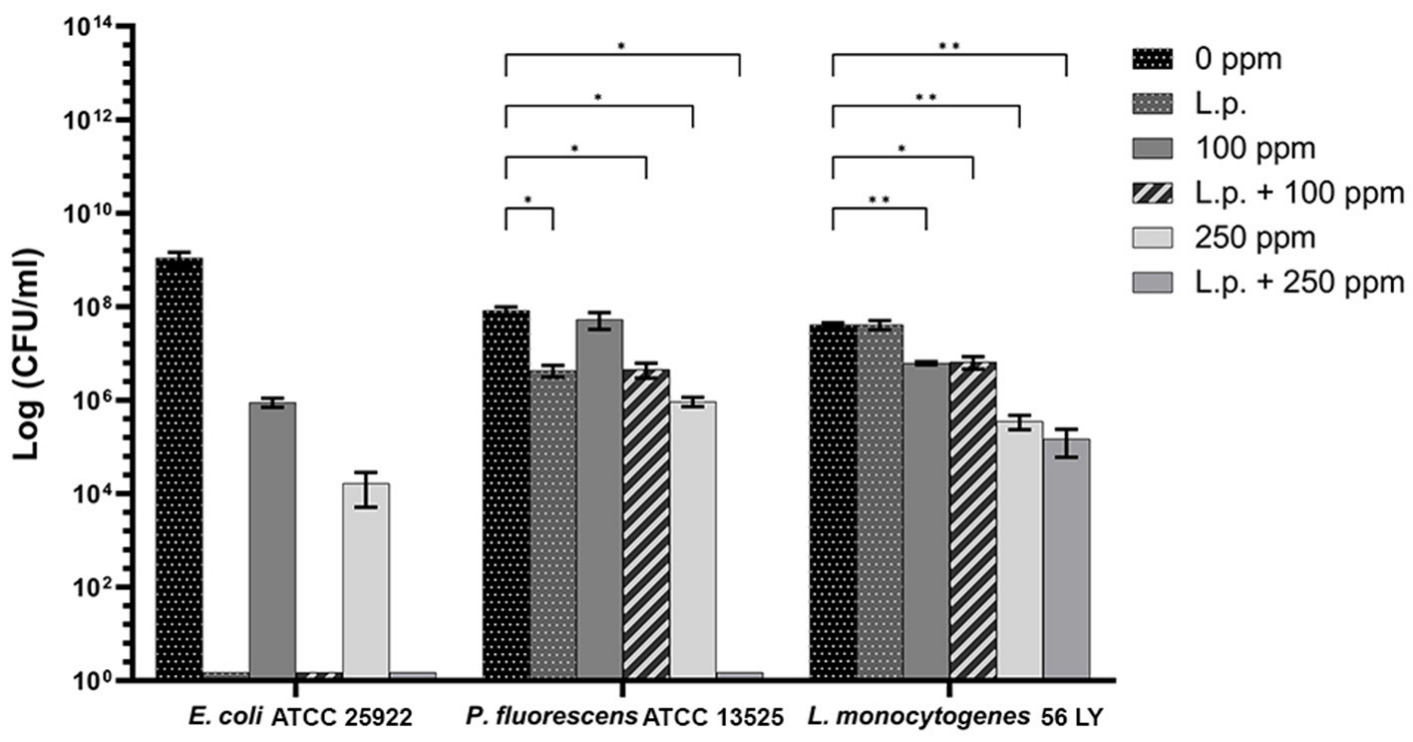
Effect of *L. plantarum* pre-formed biofilm against sessile cells of *E. coli* ATCC 25922, *P. fluorescens* ATCC 13525, and *L. monocytogenes* 56 LY grown in BHI broth (*E. coli* and *L. monocytogenes*) and TSB broth (*P. fluorescens*) and plated on BHI agar (*E. coli* and *L. monocytogenes*) and TSA agar (*P. fluorescens*). Histograms indicate the Log CFU/ml values after antimicrobial treatments: single treatments with *L. plantarum* pre-formed biofilm (*L.p*.), carvacrol 100 ppm, and carvacrol 250 ppm against each strain, and combined treatments with carvacrol 100 ppm (*L.p*. + 100 ppm) and 250 ppm (*L.p*. + 250 ppm), compared with control (0 ppm) after 24 h of incubation. Data are presented as mean ± standard deviations (SD) based on triplicates from three independent experiments. (*) *p* < 0.05, (**) *p* < 0.01, (***) *p* < 0.001, and (****) *p* < 0.0001.

**Figure 11:**
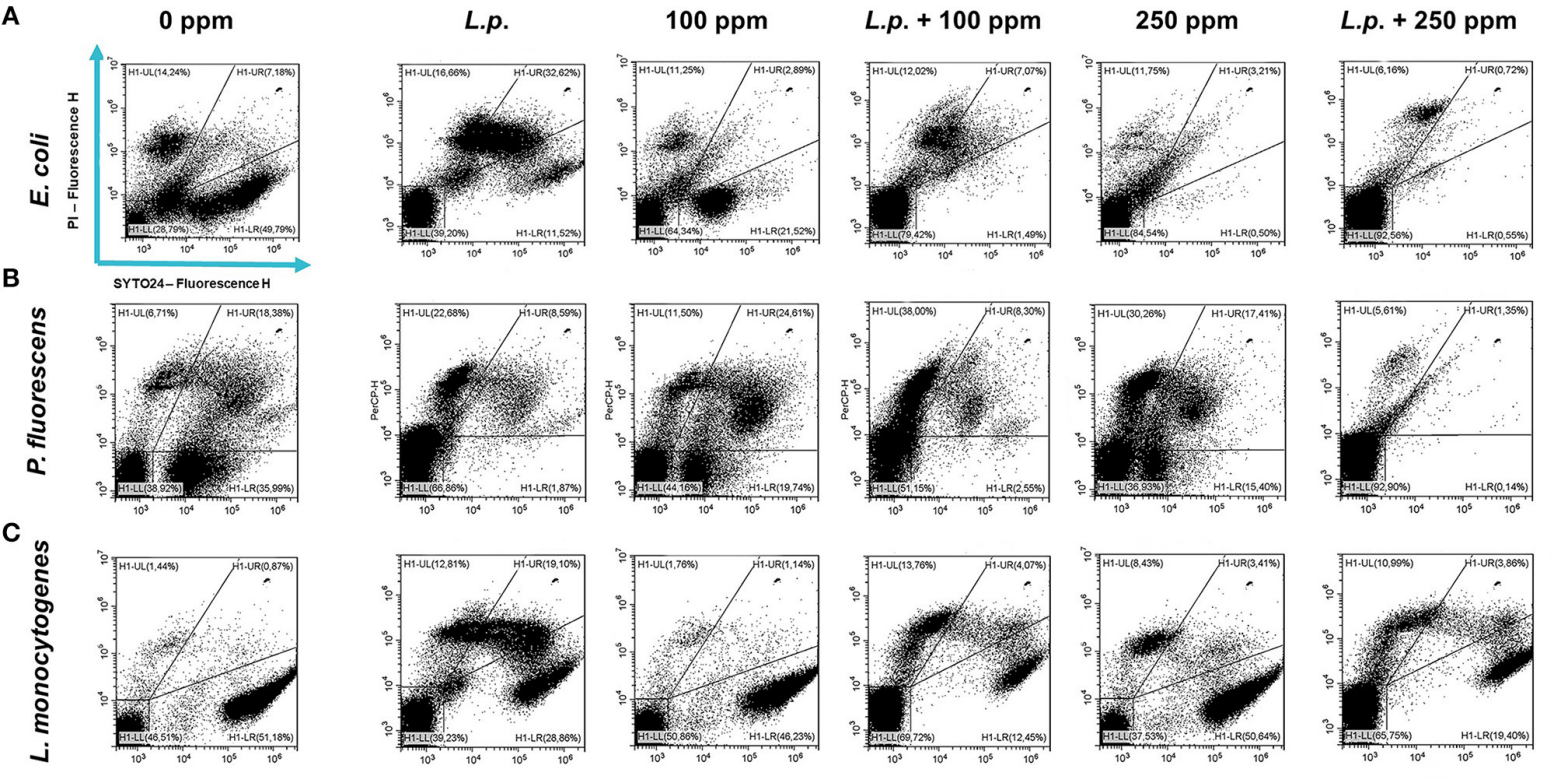
Double-staining dot plot of *E. coli* ATCC 25922 **(A)**, *P. fluorescens* ATCC 13525 **(B)**, and *L. monocytogenes* 56 LY **(C)** sessile cells grown in the presence of *L. plantarum* pre-formed biofilm, diluted in PBS (pH 7.4). The addition of *L. plantarum* pre-formed biofilm to tested strains (*L.p*.) and combined treatments with carvacrol 100 ppm (*L.p*. + 100 ppm) and 250 ppm (*L.p*. + 250 ppm), compared with control (0 ppm), carvacrol 100 and 250 ppm single treatments, after 24 h of incubation. Cells were stained with SYTO24 and PI simultaneously. H1-LL, unstained debris; H1-LR, intact cells/viable cells (SYTO24); H1-UR, injured cell population; H1-UL, permeabilized/dead cells (PI).

A total loss of culturability in *E. coli* was detected after the pre-formed biofilm *L. plantarum* monolayer application, showing an enhanced antibiofilm efficacy when the probiotic action was completed. In addition, the absence of growth was observed when *L. plantarum* +100 ppm and *L. plantarum* + 250 ppm treatments were applied. *P. fluorescens* displayed a significant reduction of 1.52 with the single pre-formed *L. plantarum* treatment (*p* < 0.05), remaining comparable to the combined *L. plantarum* + 100 ppm one (1.43). However, *P. fluorescens* cells lost their ability to grow on agar medium after combined *L. plantarum* + 250 ppm treatment. *L. monocytogenes* responded with lower sensitivity to treatments than the other strains. *L. plantarum* pre-formed biofilm alone did not result in significant logarithmic reduction, instead a significant reduction of 0.6 and 2.82 was observed in *L. plantarum* + 100 ppm (*p* < 0.05) and *L. plantarum* + 250 ppm (*p* < 0.05), respectively, compared with the control (Figure 10). The results obtained with the CV assay confirmed the culturable ones: a reduction over 80% corresponded to a loss of culturability, while a reduced antimicrobial effect on *L. monocytogenes* was detected; nevertheless, the inhibition was still proportional to the intensity of the combined treatment applied (18 and 53% inhibition in *L. plantarum* +100 ppm and *L. plantarum* + 250 ppm, respectively) (Figures 8A–C). The FCM analysis allowed us to study the efficacy of different treatments (Figure 11); as for the planktonic fraction, the combined treatment caused a bactericidal effect unlike the carvacrol single one. In *E. coli, L. plantarum* pre-formed single treatment induced cellular death which was represented by 28%, while 57% of cells were injured. A smaller fraction of 15% was still viable, even though the culture-based methods showed a complete loss of culturability. The 100 ppm single treatment induced no significant bactericidal effect (46% viable), while the combined *L. plantarum* + 100 ppm treatment displayed a significant one (68% dead cells and 29% damaged cells), expressed by a complete loss of cell viability. When comparing the combined *L. plantarum* + 250 ppm treatment with carvacrol 250 ppm alone, a substantial difference was highlighted: in the single treatment, 64% of the cells were mainly damaged, whereas, after *L. plantarum* + 250 ppm treatment, only dead cells (91%) and an increased background signal with the absence of cell viability were recorded (Figure 11A). In *P. fluorescens*, a considerable effect of the combined treatment was found. The presence of *L. plantarum* pre-formed caused 76% cell death compared with the control. When carvacrol was added to the *L. plantarum* pre-formed at the lowest concentration of 100 ppm, the bactericidal action resulted in 74% dead and 22% damaged cells. Increasing the carvacrol concentration in the combined treatment, an enhanced background signal, due to cellular debris, was observed (< 1% viable cells) (Figure 11B). Although *L. monocytogenes* explicated a lower sensitivity to the treatments, an enhanced bactericidal effect on the combined treatment was quantified. The *L. plantarum* pre-formed biofilm resulted in the following distribution of cell populations: 34% alive, 38% injured, and 28% dead, whereas in *L. plantarum* + 100 ppm and *L. plantarum* + 250 ppm, 41 and 36% cells represented dead population, respectively (Figure 11C).

## 4 Discussion

Foodborne biofilms are of particular concern in the food packaging industry, with the prevalence of some pathogens such as *L. monocytogenes, E. coli*, or spoilage (e.g., *P. fluorescens*) microbes, all found to be predominantly adherent resilient formers of biofilms on foods and food preparation and surfaces (Olanbiwoninu and Popoola, 2023). In this study, the combined bactericidal effect of carvacrol (100 and 250 ppm) and pre-formed biofilm monolayer of *L. plantarum* against foodborne pathogens (*E. coli* and *L. monocytogenes*) and spoilage (*P. fluorescens*) microorganisms, *in vitro*, was assessed. Primarily, the impact of carvacrol on *L. plantarum* was investigated to assess the carvacrol viability effect on the *L. plantarum* monolayer. The first results showed that the probiotic *L. plantarum* demonstrated strong tolerance to carvacrol. The *L. plantarum* planktonic fraction persisted viable up to 250 ppm carvacrol concentration, while the sessile fraction showed greater resistance to the antimicrobial treatment, preserving cell viability even after the 500 ppm treatment. Further tests involving carvacrol alone against *E. coli, P. fluorescens*, and *L. monocytogenes* were performed, enabling the fulfillment of two objectives: selecting the most effective carvacrol concentration against all target strains and, simultaneously, preserving the *L. plantarum* monolayer viability to enhance the overall antimicrobial effect. Additionally, the culture-based methods overestimated the effectiveness of carvacrol treatment against *E. coli, P. fluorescens*, and *L. monocytogenes*, showing a loss of culturability when the cells were still viable. FCM allows the identification of VBNC cells after using carvacrol concentrations of 250 and 500 ppm: in this state, the cells may regain culturable when favorable conditions are restored, posing a potential risk to the consumer (Jayeola et al., 2022). Moreover, the 250 ppm carvacrol concentration showed higher efficacy in inhibiting biofilm-forming adherent cells across all tested strains, while the increase in concentration (500 ppm) did not significantly enhance the antimicrobial effect. Furthermore, the lowest concentration of 100 ppm was selected to evaluate if a reduced amount of carvacrol could be utilized in a combined treatment with *L. plantarum* pre-formed biofilm, to counteract undesirable organoleptic impact and control foodborne pathogens and food spoilage biofilm (Hellebois et al., 2020; Pateiro et al., 2021). Indeed, antimicrobial resistance of biofilm-forming cells is a severe threat to the food industry (Esposito and Turku, 2023; Olanbiwoninu and Popoola, 2023). It is well-known that *Lactobacillus* species produce different exometabolites and biosurfactants with antibiofilm activity and are able to compete with pathogens for nutrients and space with different mechanisms of action (Barzegari et al., 2020). LAB can form biofilms on biotic and abiotic surfaces, acting as antagonistic effectors against various foodborne pathogens in either planktonic or biofilm mode of growth (Tatsaporn and Kornkanok, 2020; Tomé et al., 2023). Here, we found that when LAB and EOs or their components are examined individually, they exploit different effects, suggesting that their combined use can enhance the antimicrobial and antibiofilm action, improving their functioning. It is well-known that the high tolerance of LAB to lowering pH is caused by self-produced organic acids and, at low pH, the antimicrobial activity of carvacrol is enhanced due to its increased hydrophobicity, allowing it to dissolve freely in the cell membrane lipids of target bacteria (Jara et al., 2020; Cisneros et al., 2021; Sornsenee et al., 2021; Abiola et al., 2022). In addition, antimicrobial LAB-bacteriocins, which have been widely studied for their preservative properties in the food industry, improve the antimicrobial effect by reducing the carvacrol sensory impact on the food matrix (Kim et al., 2019; Simons et al., 2020; Gumienna and Górna, 2021). The combined treatment showed a more marked impact, compared with the single treatment, in the planktonic fractions and, most notably, in the sessile fractions of the tested strains. Both in *E. coli* and *P. fluorescens*, a strong growth inhibition with the combined treatment was observed. The *L. plantarum* biofilm monolayer action allowed the use of lower concentrations of carvacrol (100 ppm) to achieve substantial damage to bacterial physiology; pre-formed biofilm induced severe cellular impairment. Recognizing the cellular damage degree after treatment is crucial to avoid the resuscitation of viable but not culturable cells that could recover full pathogenicity, comparable to untreated bacterial cells in the log phase (Kan et al., 2019; Jayeola et al., 2022). This study displayed that the combined treatment produced a bactericidal effect which was not detectable by single treatments, showing mainly a bacteriostatic action. Furthermore, *L. monocytogenes* exhibited the most negligible efficacy among all strains, being the microorganism most resistant to pH changes and able to adapt in environments unfavorable to its growth (Barker and Park, 2001). Considering FCM results, it may be concluded that using carvacrol alone can determine, especially at high concentrations, stress and VBNC state induction. Nevertheless, turbidimetric assays on both planktonic and sessile cells were in agreement with the culture-based methods, underlying the need to integrate conventional microbiology methods based on culturability with advanced real-time techniques that can provide information on bacterial physiology (Fleischmann et al., 2021; Zand et al., 2021; Özel Duygan and van der Meer, 2022). Therefore, based on the findings from FCM analysis indicating that carvacrol primarily causes cellular damage rather than exerting direct bactericidal effects, the synergistic action of an *L. plantarum* monolayer combined with carvacrol presents a natural alternative to enhance the efficacy of carvacrol. This approach enables the achievement of bactericidal action, specifically against biofilm-forming cells, providing a promising alternative to be incorporated in a functionalized packaging.

## 5 Conclusion

The results of the present study confirm that the application of lactic acid bacteria (LAB) and carvacrol represents a promising solution for the natural control of food packaging pathogenic and spoilage foodborne biofilms. *L. plantarum* biofilm monolayer and carvacrol together showed an enhanced antibiofilm action, acting on the adhesion process. Indeed, the combined treatment with LAB, which create an acidic environment, fostered the interaction and dissolution of carvacrol in the cell membrane lipids of target bacteria, thus allowing its use at sub-inhibitory concentrations and achieving an increased efficacy. The FCM analysis enabled a comprehensive investigation of cell “sub-populations” distribution based on the physiological state, providing information on viability vs. culturability and highlighting the overestimation of the treatment success if considering the culture-based approaches only. Further studies are needed to test the antibiofilm combination against other pathogenic bacteria. Furthermore, it is crucial to perform *in vivo* analysis, evaluating the impact of LAB biofilm monolayer plus carvacrol on product shelf-life and characteristics, by enclosing them within a matrix or inert material (e.g., polymers). Considering the expansion of the functional food market over the decades due to increased “green consumer” demand for natural, nutritional, and healthy food products, it will be essential to implement food packaging and design successful delivery systems for such bioactive compounds.

## Data availability statement

The raw data supporting the conclusions of this article will be made available by the authors, without undue reservation.

## Author contributions

VP: Conceptualization, Data curation, Formal analysis, Investigation, Methodology, Software, Visualization, Writing – original draft, Writing – review & editing. LD: Conceptualization, Data curation, Formal analysis, Investigation, Methodology, Software, Visualization, Writing – original draft, Writing – review & editing. MC: Conceptualization, Investigation, Methodology, Writing – review & editing. CN: Methodology, Writing – review & editing. RB: Methodology, Validation, Writing – review & editing. LG: Funding acquisition, Writing – review & editing. AB: Conceptualization, Funding acquisition, Investigation, Methodology, Project administration, Resources, Supervision, Validation, Visualization, Writing – original draft, Writing – review & editing.
