# Supplementary figures and images for: Lactiplantibacillus plantarum monolayer enhanced bactericidal action of carvacrol: biofilm inhibition of viable foodborne pathogens and spoilage microorganisms
Source: Front Microbiol. 2023 Nov 22;14:1296608. doi: 10.3389/fmicb.2023.1296608 (PMC10703393; doi:10.3389/fmicb.2023.1296608)

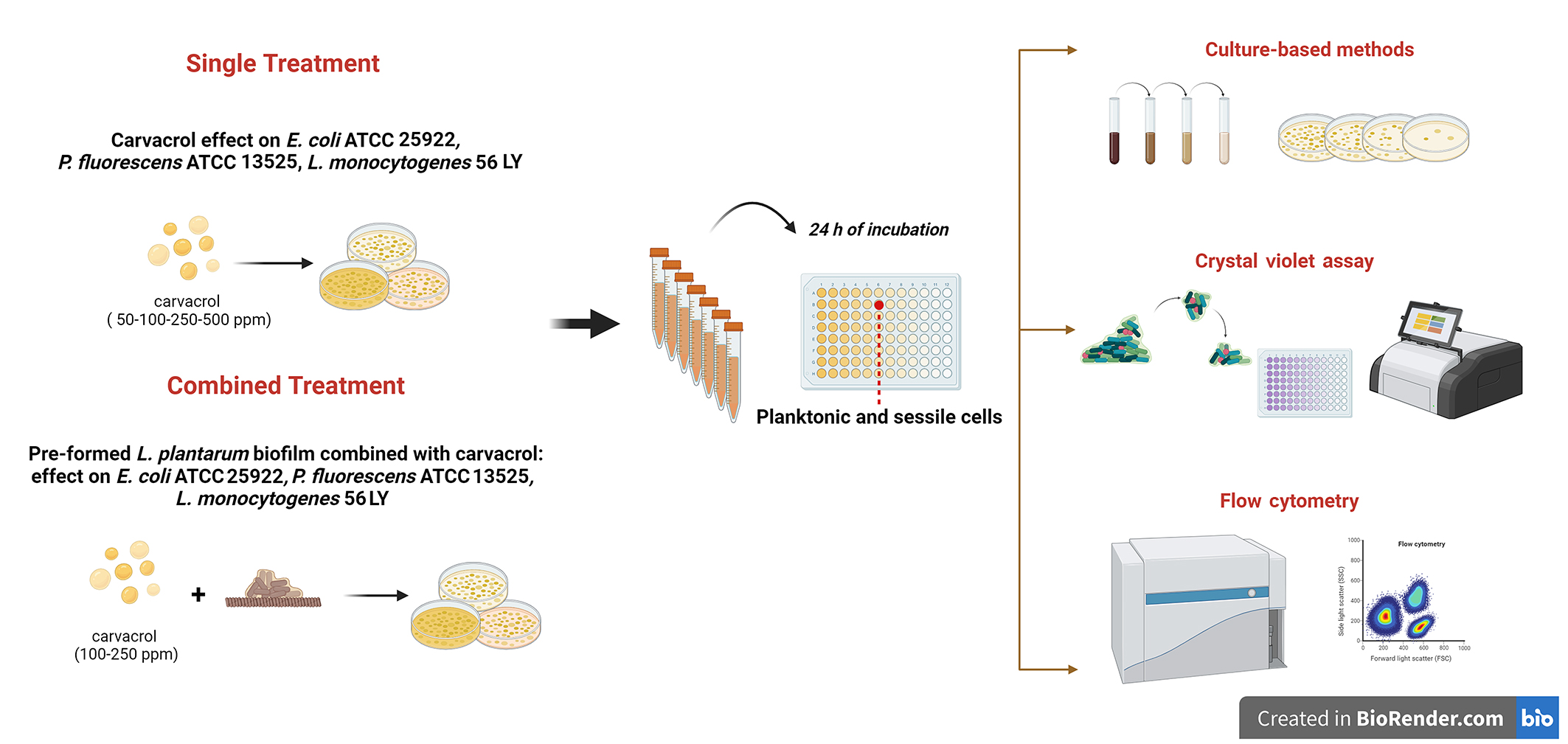

Supplement: Supplementary file 1 [file Image_1.TIF]
